# Supplementary material for: Fast, Scalable, Bayesian Spike Identification for Multi-Electrode Arrays
Source: PLoS One. 2011 Jul 20;6(7):e19884. doi: 10.1371/journal.pone.0019884 (PMC3140468; doi:10.1371/journal.pone.0019884)
Supplement: Text S1 — Supplementary appendix describing additional methodological details. (PDF) [file pone.0019884.s001.pdf]

SUPPLEMENTARY INFORMATION

A Symbols and quantities used in the text

The following symbols describe "internal" variables in our discussion:
N: Number of electrodes times number of time samples in an event; in our experiment $N = 30 \times 32 = 960$.
x, y: Integer coordinates giving electrode address in the array.
$\bar{x}$, $\bar{y}$: Interpolated coordinates giving the barycenter of an event (Fig. 2A).
x_0, y_0: Address of "leader" electrode for an event.
$d(x, y)$: Distance function used in OPTICS.
$V_{x,y}(t)$: Electric potential trace in an event. $\mathbf{V}$: Same N quantities regarded as a vector.
μ_i: Index specifying the unit identity of the ith spike in an event.
t_i: Time of the ith spike in an event, relative to the start of the event.
A_i: Amplitude rescaling factor of the ith spike in an event..
$F_{\mu;x,y}(t)$: Template waveform. $\mathbf{F}_{\mu,t_i}$: The shifted waveform $F_{\mu,x,y}(t - t_i)$ regarded as a vector.
$\delta\mathbf{V} = \mathbf{V} - \sum_i A_i \mathbf{F}_{\mu,t_i}$: Residual after subtracting spikes.
$P_{\text{noise}}(\mathbf{V})$: Distribution of noise.
$P(\mathbf{V}|\{\mu_i, t_i, A_i\})$: Likelihood of a set of spikes.
$P(\mu_1, t_1, A_1)$: Prior probability density for a spike of type μ_1 to occur at time t_1 with amplitude A_1 times that of the template.
$\delta_{x,x'}$: Kronecker delta.
C: Covariance matrix of noise distribution. G: Inverse covariance (SI Sect. G.1).

In addition, the analysis requires numerical values of some quantities that are "external" to the analysis (i.e. preparation- and apparatus-dependent). Here we list these and sketch how they are found:
$\delta t = 0.1$ ms: Inverse of sampling rate.
$V^*_{\text{spike}} = -40\,\mu$V: Amplitude threshold for a spike event, determined by requiring that spikes extend beyond noise. It is approximately four times the standard deviation of the data.
$V^*_{\text{noise}} = -30\,\mu$V: Amplitude threshold for a noise clip.
$V^*_{\text{trust}} = -44\,\mu$V: Amplitude threshold for a trusted cluster, set slightly more stingently than V^*_{spike}.
η, τ and ξ: Parameters describing the empirical noise distribution, obtained by the method in SI Sect. G.1.
γ_μ, σ_μ: Mean and standard deviation, respectively, of the distribution of A factors for cluster μ. Prior to the first fitting pass, these are estimated by examining the events that led to cluster μ; after the first pass they are obtained by examining all fits of cluster μ to data.
r_μ: Average firing rate of unit μ. Prior to the first fitting pass, these are estimated from the number of events in cluster μ; after the first pass they are obtained from the number of times μ was fit.
Sampling rate = 10 kHz.
High-pass filter threshold = 200 Hz.
Event duration = 3.2 ms.
Cropped neighborhood size = 9 channels.
Data subset for clustering = 4 non-consecutive 30 s clips.
Upsampling factor = 5.
Noise threshold used in clustering distance function = $-15\,\mu V$
Template cross-correlation above which clusters are candidates for merging = 50%.

B Outline of procedure

Bold indicates significant human intervention and judgment.

A. Preprocess

- High-pass filter.
- **Examine data and set V^*_{spike} and V^*_{noise}.**
- Extract spike events and noise clips.
- Obtain spatial covariance of noise and use to spatially whiten all data.
- Identify small subset of data drawn uniformly from entire experimental run for clustering/template building.
- Find temporal covariance of spatially whitened noise clips and summarize by η, τ.

B. Cluster and build templates

- Segment each spike event, sometimes splitting it into multiple elementary events.
- Classify elementary events by leader channel. Separately for each leader channel do:
  - Upsample, temporally align to peak, crop to neighborhood of leader.
  - **Choose V^*_{mask} and use to mask out remaining low-amplitude channels.**
  - Sort events using OPTICS.
  - **Cut into clusters.**
- Repeat twice:
  - Examine all pairs of clusters for similarity and display similar pairs to user.
  - **Manually determine which pairs are duplicates and merge them.**
  - **Manually examine each cluster and determine which are mixtures. For each mixture manually select the most divergent channel.**
  - **Compute PCA on most divergent channel and manually cut on most divergent feature to split the cluster.**
- For each cluster:
  - Upsample all exemplars in each cluster and find consensus template via pointwise median.
  - **Discard clusters whose pointwise median and mean are significantly different.**
  - **Discard clusters that seem to involve an axon, that seem to reflect simultaneous firing of two more elementary waveforms, or whose total population is small.**
  - Obtain distribution of rescaling factors A and summarize by mean and variance.
  - Obtain estimated mean firing rate.

C. Fit spikes

- Make two passes:
  - For each spike event,
    - Maximize eqn. 5 to ID the main spike type.
    - Minimize mean-square deviation to find the amplitude and arrival time, then subtract scaled template.

  * If the log-posterior has increased, repeat.
- If the signal, minus all fit spikes, exceeds threshold flag the fit as incomplete. Then proceed to next spike event.

- After first pass, update mean, variance, and rate estimates for each cluster.
- **Recluster all the residuals of incomplete fits, then make second pass.**

D. Choose trusted templates

- For each template:
  - **Manually examine histogram of the fit values of A and set a threshold between true fits and noise fits.**
  - **Flag template as untrustworthy if the histogram is too nongaussian or if the true fit peak significantly overlaps the noise fit peak (or extends below V^{*}noise).**
  - **Also untrustworthy if time series of fit A values, or firing rate, is not stationary.**
  - **Check spike time autocorrelations; if there is no refractory hole (too many refractory violations), then flag the cluster as untrustworthy.**
- **Check spike time cross-correlations; if there is a refractory hole between two templates then consider merging those templates' spike trains.**

C Stimulus

We alternated between presenting two kinds of stimuli. Each stimulus was an illumination pattern consisting of a 15×15 checkerboard. The checks were illuminated with green light in 33 ms frames. (a) In the "white noise" stimulus, each check on each frame was drawn independently from a binary (2-level) distribution of intensities. (b) In the "correlated noise" stimulus, each check was correlated with its neighbors, with exponential correlation falloff in space and time and the same mean illumination as in (a). The space constant for correlation falloff was one check; the time constant was 33 ms.

D Preprocessing

Our array consisted of two groups of 30 electrodes each, separated by 500 μm (Multi Channel Systems MCS GmbH, Reutlingen, Germany); we expect signals on the two groups to be independent so we analyzed the data from just one group at a time. Following high-pass filtering and spatial whitening (see main text), spikes were identified by one of two methods depending on whether they were to be used in the clustering or fitting stage of the algorithm:

Clustering All time points at which the potential crossed $V^{*}_{\text{spike}} = -40\,\mu$V were identified and then divided into segments as described in the main text. Spikes were then taken as fixed spatial and temporal neighborhoods of the peak within each segment. The segmenting was performed with a modified version of the breadth-first search algorithm [1], which identifies all connected components of a graph.

Fitting We identified periods of time throughout which at least one channel remained more negative than V^{*}_{spike} (though not necessarily the same channel throughout the period). The overall peak within each such period was identified and the spike taken as a fixed temporal neighborhood of the peak, keeping all channels. The advantage of this method over the segmentation method used prior to clustering is increased speed. However, we expect that it will break down for large arrays, where there are very few "silent" periods with no channel below threshold. In this case the segmentation method can still be used.

E Clustering method

E.1 Data preparation

The procedure for data preparation described in the Methods section of the main paper leads to a set of spike events. These were interpolated to achieve a virtual sampling rate of 50 kHz and then temporally aligned to place the peak (absolute minimum) of the waveform at a common position. The OPTICS algorithm was then applied to the upsampled, aligned, events.

E.2 OPTICS algorithm

We wished to identify clusters of spike events that corresponded to different neural units. Each cluster was then reduced to one representative template. Automated clustering is a hard problem which often leads to unsatisfying results. We chose to use an algorithm that does not produce a clustering of the data, but instead creates an augmented ordering of the extracted spikes. The ordering put similar spikes together, therefore making clusters easy to identify. The advantage of this method was that we kept full control over the actual clustering process and could easily understand the quality of our clusters.

The ordering algorithm, called OPTICS (*O*rdering *P*oints *T*o *I*dentify the *C*lustering *S*tructure) [2], chooses a random starting point and generates a walk through the data points (which are our extracted spike events) which tries to follow a route of decreasing density. Therefore, points that are densely packed together end up close to each other in the ordering. At each iteration, OPTICS assigns to each point p a "reachability distance" to the current point, defined by an integer parameter k. If the current point is not one of the k nearest neighbors of p, then the reachability distance is just the conventional distance to p (our choice of metric was described in the main text). Otherwise, the reachability distance is the distance from p to its kth nearest neighbor. Thus, points with lowest reachability distance tend to be both near to the current point and located in regions of high density. The algorithm steps to the unvisited point with lowest reachability distance, and iterates. Because points in high density regions are visited first, OPTICS avoids following a single chain of points (the "single link effect").

E.3 Graphical user interface

The subset of the data was ordered with the OPTICS algorithm and then presented to the user in graphical form (Fig. 2D). The user then cut the desired clusters by scanning linearly through the data. The presentation of the data was done in such a way that the user could recognize contiguous clusters as horizontal stripes within the full set of data. This was done by presenting the voltage traces of all channels of a particular spike along a vertical column of pixels. Therefore, similar spikes produced similar vertical color coded patterns that were visible as horizontal stripes.

E.4 Merging, splitting, and discarding clusters

This section describes some machine-assisted human judgement steps that we found were essential for accurate spike identification. We arranged that the human would never be comparing each template to all others, so that the effort would scale roughly linearly with array size.

E.4.1 Merging

The spike events used for clustering were segregated into groups based on the electrode number of their leader (peak) channel; OPTICS was used separately on each group to separate it into clusters. Tentative templates were then found for each cluster. To see if any of these were duplicates, we calculated the cross-correlations of all template pairs at all relative time shifts, and maximized over time shift. Our code flagged for scrutiny those pairs with maximal cross-correlation greater than 50%, and displayed such pairs to the

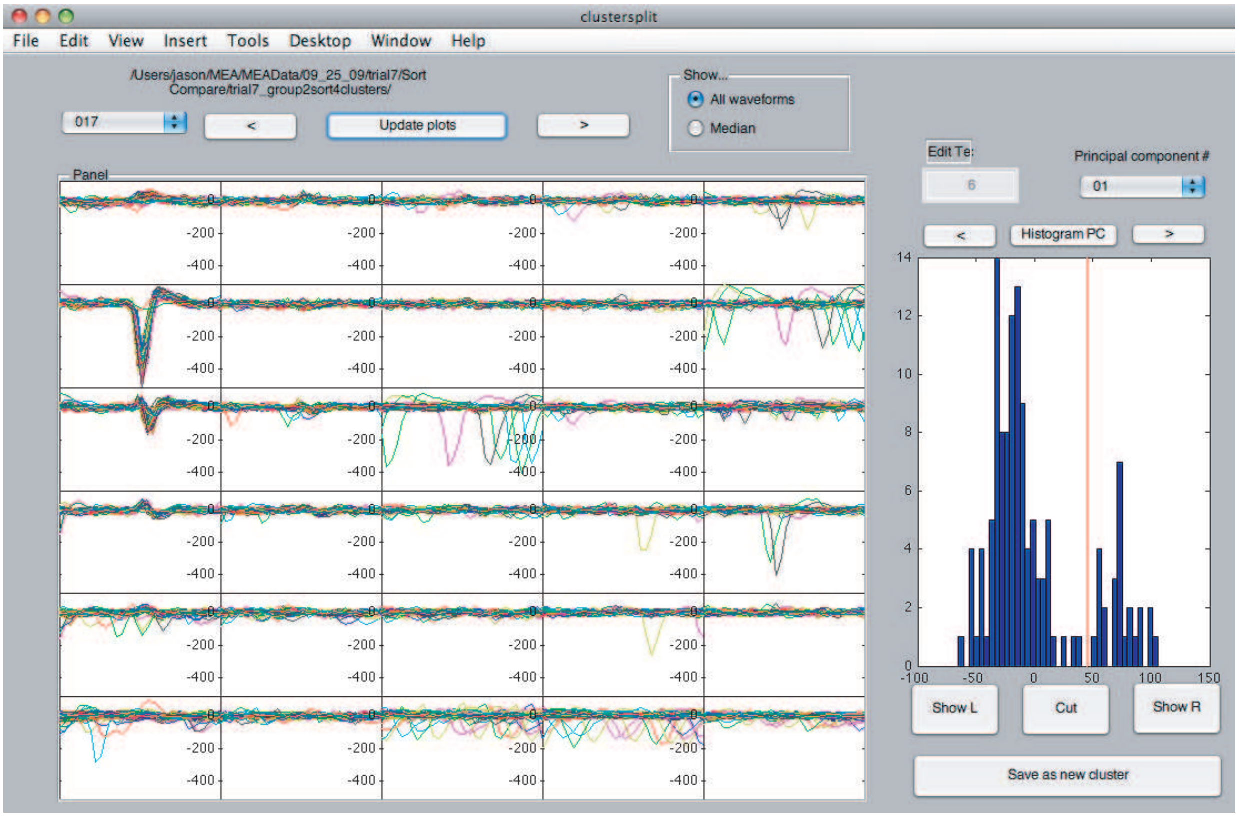

Figure S1: The graphical user interface used for splitting clusters. The user selects a channel (one of the left panels) which appears multimodal; in this case, the first channel in the second row. A principal component analysis is performed on the waveforms of just that channel and a histogram of the weights of each component is displayed (right panel). The user has indicated a threshold (vertical red line) at which the software will split the cluster into two.

human operator. The operator could then quickly identify the roughly 10% of presented pairs that were the same. The code then merged the corresponding sets of events into a single cluster.

E.4.2 Splitting

In some cases, the OPTICS approach yielded putative clusters that were actually composites of two different waveforms. We wrote a second GUI to overlay all the traces of a cluster and display to the human operator (Fig. S1). Compositeness was generally visually obvious. The operator had the option to accept the cluster as-is, or split it. In the latter case, the operator selected an electrode whose trace was particularly bimodal, and the code computed a principal component analysis on the waveforms of the events restricted to that electrode. Then the code displayed histograms of the projections of the events onto each principal component and allowed the operator to select a threshold on whichever histogram showed the best separation. It can happen that, after splitting a cluster, one of the subcomponents turns out to be identical to some existing cluster. To address this possibility, the merging step in SI Sect. E.4.1 was repeated one additional time.

E.4.3 Discarding

Finally, all clusters emerging from the preceding steps were visually inspected and a few were discarded. (The data in this paper led us to discard about 15% of the clusters.) Typical grounds for discarding a cluster were that: (1) Its waveforms were the same as those of another cluster, plus an axon trace that did not interest us. (2) It was a superposition of two waveforms, each of which corresponded to another existing cluster. (3) It contained very few events, leading to poor agreement between its mean and median waveforms (see Fig. 5A for an example of successful agreement).

E.4.4 Second pass

Some new clusters were found after the first pass through the full dataset. The steps in SI Sects. E.4.1–E.4.3 were repeated with the resulting, augmented set of clusters prior to performing the second pass.

F Template generation

To obtain good fits, it is essential to have template waveforms that accurately capture the intrinsic shapes and variability of the spikes. For example, some prior work did not allow for variation in spike amplitude and hence inferred very small likelihood, even for the correct template. We addressed this issue by fitting an amplitude rescaling factor A, and we argued that most intrinsic spike variation is captured by this one factor.

Spikes from a single unit may also vary slightly in duration, which can also lead to spuriously low likelihood. Unfortunately, unlike variation in A, these variations cannot be expressed as linear transformations on the templates. Instead, we created two extra versions of each of our templates, one stretched temporally by 5%, and another squeezed by the same amount. The fitted spike times for all three versions were merged to give a single spike train for the cluster.

G Noise characterization

G.1 Inferring parameters

As noted in the main text, our empirical noise distribution has a 2-point correlation function that is approximately translation-invariant in space and time, diagonal in space, and exponential in time. Dropping the trivial spatial indices, then

$$\mathsf{C}(t,t') = \eta \mathrm{e}^{-|t-t'|/\tau}. \tag{S1}$$

The inverse of this infinite matrix is an infinite, symmetric, translation-invariant matrix. To confirm that it is the inverse of the band-diagonal matrix in Eqn. 2, multiply the two matrices:

$$\begin{aligned}\sum_{t''} \mathsf{C}(t,t'')\mathsf{C}^{-1}(t'',t') &= \sum_{t''} \left[\eta \mathrm{e}^{-|t-t''|/\tau}\right] \left[\frac{1}{\eta(1-\xi^2)} \left((1+\xi^2)\delta_{t'',t'} - \xi\delta_{t'',t'+\delta t} - \xi\delta_{t'',t'-\delta t}\right)\right] \\ &= \frac{1}{1-\xi^2}\left((1+\xi^2)\mathrm{e}^{-|t-t'|/\tau} - \xi\mathrm{e}^{-|t-t'-\delta t|/\tau} - \xi\mathrm{e}^{-|t-t'+\delta t|/\tau}\right)\end{aligned}$$

We have dropped the spatial indices in Eqn. 2 since they give a trivial factor of the identity matrix. The claim is that the above expression equals the identity matrix in time, $\delta_{t,t'}$. Taking $t = t'$, and using $\delta t > 0$

and $\mathrm{e}^{-\delta t/\tau} = \xi$,

$$\begin{aligned}\sum_{t''} \mathsf{C}(t,t'')\mathsf{C}^{-1}(t'',t) &= \frac{1}{1-\xi^2}\left(1+\xi^2-2\xi\mathrm{e}^{-\delta t/\tau}\right)\\ &= \frac{1}{1-\xi^2}(1-\xi^2)\\ &= 1.\end{aligned}$$

Next, consider $t > t'$. Since δt is the sampling time, we also have $t - t' \geq \delta t$ and can therefore drop all absolute values:

$$\begin{aligned}\sum_{t''} \mathsf{C}(t,t'')\mathsf{C}^{-1}(t'',t') &= \frac{1}{1-\xi^2}\left((1+\xi^2)\mathrm{e}^{-(t-t')/\tau} - \xi\mathrm{e}^{-(t-t'-\delta t)/\tau} - \xi\mathrm{e}^{-(t-t'+\delta t)/\tau}\right)\\ &= \frac{\mathrm{e}^{-(t-t')/\tau}}{1-\xi^2}\left(1+\xi^2-\xi\mathrm{e}^{\delta t/\tau}-\xi\mathrm{e}^{-\delta t/\tau}\right)\\ &= \frac{\mathrm{e}^{-(t-t')/\tau}}{1-\xi^2}\left(1+\xi^2-1-\xi^2\right)\\ &= 0.\end{aligned}$$

A similar derivation, flipping the signs in the exponentials, shows that the off-diagonal terms vanish for $t < t'$, completing the proof.

The parametric form of C in Eqn. S1 is not an exact description of the data, of course, nor is any Gaussian distribution exactly correct. However, this form has a key feature: Its inverse Eqn. 2 is *local* in time (it mixes each sample only with its nearest neighbors), and hence maintains the distinctness of spikes occurring at different times. To use this formula, we now need to find numerical values of the parameters η and τ that best represent the data. To do this, we compute a likelihood function, the probability that the actual noise data *would* have been observed, had they actually been distributed according to a member of the parametric family.

Suppose we have M noise samples $\mathbf{V}_1, \ldots, \mathbf{V}_M$, each with a very large number L of components and distributed as

$$P_{\mathsf{G}}(\mathbf{V}) = (2\pi)^{-L/2}(\det \mathsf{G})^{1/2}\mathrm{e}^{-\mathbf{V}^{\mathrm{t}}\mathsf{G}\mathbf{V}/2}.$$

The log likelihood is then

$$-\frac{LM}{2}\log(2\pi) + \frac{M}{2}\mathrm{Tr}\,\log\mathsf{G} - \frac{1}{2}\sum_i^M \mathbf{V}_i^{\mathrm{t}}\mathsf{G}\mathbf{V}_i.$$

Its first variation is $(M/2)\mathrm{Tr}\,\mathsf{G}^{-1}\delta\mathsf{G} - (1/2)\sum_i \mathbf{V}_i^{\mathrm{t}}(\delta\mathsf{G})\mathbf{V}_i$.

We wish to maximize the likelihood over the restricted class of distributions with

$$\mathsf{G} = \begin{bmatrix} P & Q & & \\ Q & P & Q & \\ & Q & P & Q \\ & & Q & \ddots \end{bmatrix}; \qquad \mathsf{G}^{-1} = \eta \begin{bmatrix} 1 & \xi & \xi^2 & \cdots \\ \xi & 1 & \xi & \cdots \\ \xi^2 & \xi & 1 & \cdots \\ \vdots & & & \ddots \end{bmatrix},$$

where $P = \frac{1}{\eta}\frac{1+\xi^2}{1-\xi^2}$, $Q = \frac{1}{\eta}\frac{\xi}{\xi^2-1}$. To do this we now set the first variations of log-likelihood, as parameters P and Q are varied, equal to zero.

We are interested in two kinds of variation: First, varying P leads to

$$0 = \frac{M}{2}\mathrm{Tr}\,\mathsf{G}^{-1}\frac{\partial\mathsf{G}}{\partial P} - \frac{1}{2}\sum_i^M \mathbf{V}_i^{\mathrm{t}}\frac{\partial\mathsf{G}}{\partial P}\mathbf{V}_i,$$

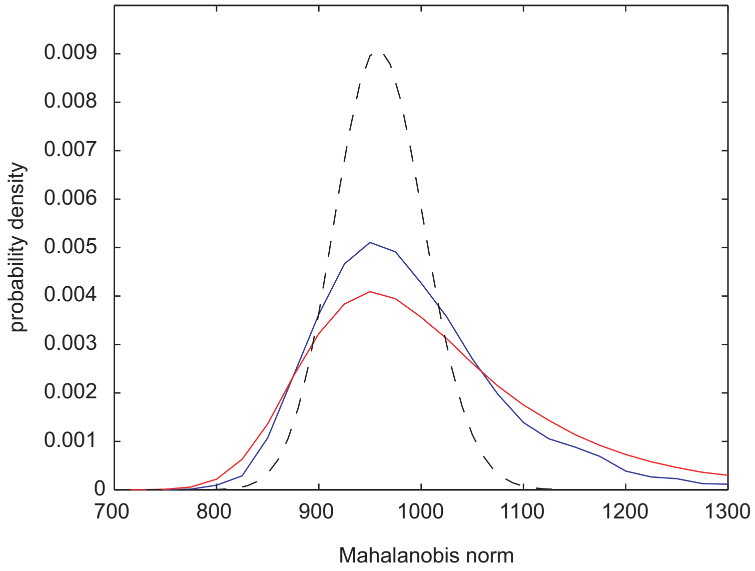

Figure S2: Top to bottom: *Dashed,* chi square probability distribution with $N = 960$ degrees of freedom. *Blue,* distribution of Mahalanobis norms of noise clips, computed using the empirical metric Eqn. 2. *Red,* corresponding distribution for residuals left after removing all identifiable spikes from spike events. All three curves peak at norm ≈ 960, the assumed number of independent degrees of freedom. The two experimental distributions are similar, supporting our claim that we successfully found most above-threshold spikes. However, both solid curves are broader than the dashed, pure-Gaussian prediction; the discrepancy reflects the fact that even "noise" traces actually include some subthreshold spikes.

or $\eta = \frac{1}{L}\langle \mathbf{V}^2 \rangle$. Here the expectation value refers to an average over noise samples (and over the electrode address, which has been suppressed from the notation).

Second, varying Q gives

$$0 = \frac{LM}{2} 2\eta\xi - \frac{1}{2}\sum_{t,i} 2V_i(t)V_i(t+1),$$

or $\xi = \frac{1}{L\eta}\langle \sum_t V(t)V(t+1) \rangle$. Evaluating the indicated expected values thus gives us numerical values for η and ξ (or equivalently τ), and hence P and Q.

G.2 Evaluation

The method just outlined gave the noise model evaluated in Fig. 6B. A different test looks not at the one-point marginal distribution, but instead at the distribution of the total "Mahalanobis norm" $\mathbf{V}^t \mathsf{C}^{-1} \mathbf{V}$ evaluated over all noise clips [3, 4]. If the noise were truly Gaussian, this quantity would be the sum of independent normal deviates and so would follow the chi-square distribution with N degrees of freedom. SI Fig. S2 compares these quantities. Although the two distributions peak at the same place, the empirical distribution of Mahalanobis norms is broader on its high end than the chi square distribution [4], in part because our noise clips still contain subthreshold spikes ("spikelets"). The presence of spikelets does not interfere with our algorithm's ability to identify the higher-amplitude spikes of interest to us. However, they can have large Mahalanobis norms because, although low in peak amplitude, they have many samples that deviate from the true noise in a correlated way.

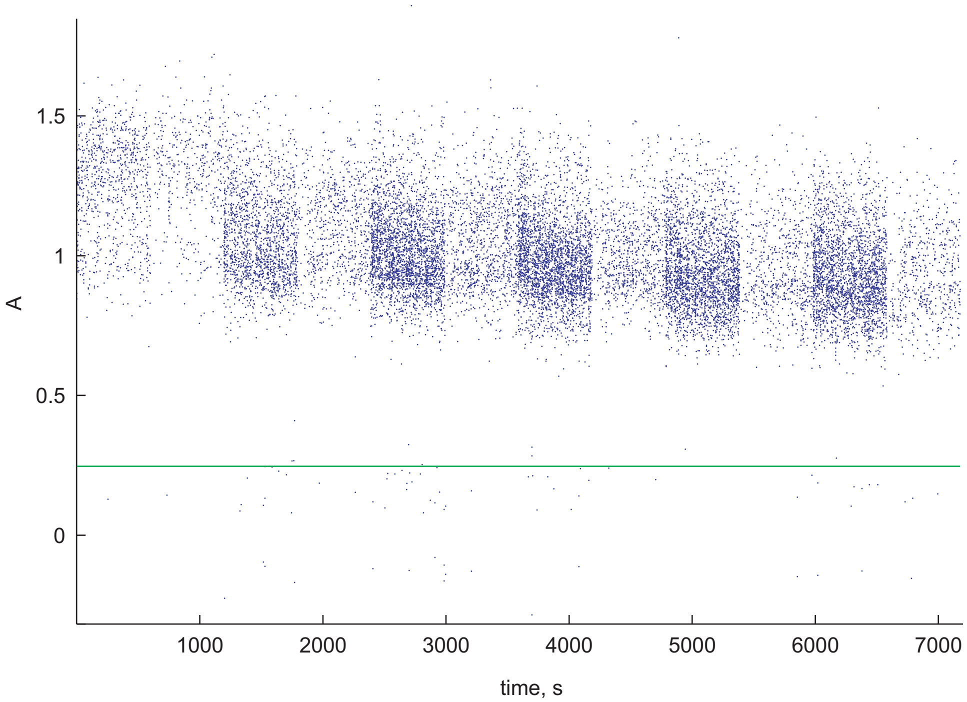

Figure S3: After an initial settling-in period, most cells behave uniformly throughout the experiment. Each fit spike for a particular template gave rise to a dot denoting its arrival time and amplitude scaling factor A. The vertical band structure shows the presented visual stimulus alternating every 10 min between the two forms described in SI Sect. C. (The higher firing rate corresponds to the correlated stimulus.) For the cluster in question, a peak amplitude 44 μV corresponds to an A value shown as the solid green line. Dots below this line were presumably noise fits and were rejected as described in Methods, step 4. Interestingly, not only the frequency, but also the range of amplitudes depends on the stimulus type.

Shoham et al. noted that a multivariate t-distribution generated better agreement with the observed distribution of Mahalanobis distances than the Gaussian noise model used in the present work [4]. Certainly one can always improve agreement by using a family of distributions with an additional fit parameter. But the Gaussian has a key feature that the t-distribution of [4] lacks: It factorizes into independent distributions for each dimension in the decorrelated data. Combined with the locality of our C^{-1} matrix, this property ensures that a sample containing multiple temporally separated spikes will have a likelihood that is essentially the product of independent factors for each of the spikes. This implies that when we fit spike #1, the contribution to log-likelihood from other spikes *cancels* from numerator and denominator of the likelihood ratio described in the main text, allowing the algorithm to focus on one spike at a time. For this reason, and because our performance metrics show that our method works well, we elected to use the computationally more efficient Gaussian noise model.

H Evaluation of clusters after spike fitting

To decide which fitted clusters were "trusted," we asked:

*(1)*Does the residual of one-spike events, after removal of the fit spike, look clean (see Fig. 6C)?

*(2)*Is the histogram of fit scale factors approximately a Gaussian, centered at $A \approx 1$, and well separated

from any noise peak?

*(3)*Does that histogram mostly lie above an A value corresponding to V^*_{trust}?

*(4)*Does the time course of fit values of A exhibit significant drift over the duration of the experiment? If so, this could indicate that a neuron is dying or otherwise not reliable, or it could simply define a range of times in which the neuron is stable (see SI Fig. S3).

*(5)*Similarly, does the unit corresponding to this template abruptly stop (or start) firing altogether somewhere in the middle of the experiment?

*(6)*Do the interspike intervals for this template display a "hole" corresponding to a neural refractory period?

I Relation to other work

Several authors have pointed out limitations to the Gaussian model of noise, for example [5, 6, 4]. Some care must be taken when evaluating these statements, however. In many prior works, spike waveforms were regarded as generated by a mixture of Gaussian distributions on feature space. That is, all spike variation was regarded as arising from (a) the discrete choice of which spike μ fired, and (b) an additive, Gaussian-distributed variation with covariance Σ_μ depending on μ. This approach requires one to fit a very large number of parameters (a separate covariance matrix for every cluster), and so becomes impractical with many clusters and many features. It also makes no distinction between extrinsic noise (e.g. in the amplifiers) and *intrinsic* spike variation, which we found was best modeled as *multiplicative*.

In contrast, our work models spike variation as arising from (a′) the discrete choice of μ, (b′) a single multiplicative factor A, with mean and variance depending on μ, and (c′) universal, additive noise characterized by two numbers η and τ. Thus our claim that (c′) may be modeled as a correlated Gaussian is not directly contradicted by others' observations, and indeed appears reasonable in the light of Fig. 6B. It is true that other tests show that our noise clips are not literally Gaussian-distributed (SI Fig. S2), but we have argued that this discrepancy reflects unresolved, subthreshold spikes, and does not impair our ability to identify larger amplitude spikes. Indeed we have presented several tests showing that we do have reliable spike identification.

Moreover, Harris et. al. have suggested that some of the discrepancy in the Mahalanobis norm distribution can be attributed to spike overlaps [6], which our method can resolve. Both Gaussian and non-Gaussian mixture models are unable to handle overlaps without special modification. Indeed, it was the desire to handle such overlaps that led us to our approach (see SI Sect. G.2).

Some prior authors have also attributed their observed non-Gaussian behavior in part to variation in spike amplitude, for example during a burst [5]. Our method handles such variation by adjusting the fit amplitude prior to subtracting the spike (Fig. 4B).

Finally, our approach to the problem, which involves separating clustering from fitting, made it possible for us to use a clustering method that made no use whatever of any mixture model, Gaussian or not. Unlike some prior approaches [7], our segmentation method makes it unnecessary to have isolated ("clean") examples of every spike type.

References

[1] Knuth DE (1997) The Art Of Computer Programming, volume 1. Boston: Addison-Wesley, 3rd edition.

[2] Ankerst M, Breunig MM, Kriegel HP, Sander J (1999) OPTICS: Ordering points to identify the clustering structure. SIGMOD Rec 28: 49–60.

[3] Pouzat C, Mazor O, Laurent G (2002) Using noise signature to optimize spike-sorting and to assess neuronal classification quality. J Neurosci Methods 122: 43–57.

[4] Shoham S, Fellows MR, Normann RA (2003) Robust, automatic spike sorting using mixtures of multivariate t-distributions. J Neurosci Methods 127: 111–22.

[5] Fee MS, Mitra P, Kleinfeld D (1996) Automatic sorting of multiple unit neuronal signals in the presence of anisotropic and non-gaussian variability. J Neurosci Methods 69: 175–188.

[6] Harris KD, Henze DA, Csicsvari J, Hirase H, Buzsáki G (2000) Accuracy of tetrode spike separation as determined by simultaneous intracellular and extracellular measurements. J Neurophysiol 84: 401–14.

[7] Segev R, Goodhouse J, Puchalla J, Berry MJ (2004) Recording spikes from a large fraction of the ganglion cells in a retinal patch. Nature Neuroscience 7: 1154–61.
